# Neglected burden of injuries in Ethiopia, from 1990 to 2019: a systematic analysis of the global burden of diseases study 2019

**DOI:** 10.3389/fpubh.2023.1149966

**Published:** 2023-05-22

**Authors:** Tezera Moshago Berheto, Mathilde Sengoelge, Sebsibe Tadesse, Shimelash Bitew Workie, Gizachew Tessema, Solomon Tessema Memirie, Shikur Mohammed, Fentabil Getnet, Ally Walker, Mohsen Naghavi, Awoke Misganaw

**Affiliations:** ^**1**^National Data Management and Analytics Center for Health, Ethiopian Public Health Institute, Addis Ababa, Ethiopia; ^2^Department of Global Public Health, Karolinska Institutet (KI), Stockholm, Sweden; ^3^School of Population Health, Faculty of Health Sciences, Curtin University, Perth, WA, Australia; ^4^College of Health Sciences, Addis Ababa University, Addis Ababa, Ethiopia; ^5^Institute of Health Metrics and Evaluation, University of Washington, Seattle, WA, United States

**Keywords:** disability-adjusted life years, global burden of diseases, injuries, interpersonal violence, self-harm, transport injuries, years lived with disability, years of life lost

## Abstract

**Background:**

The 2030 agenda for sustainable development goals has given injury prevention new attention, including halving road traffic injuries. This study compiled the best available evidence on injury from the global burden of diseases study for Ethiopia from 1990 to 2019.

**Methods:**

Injury data on incidence, prevalence, mortality, disability-adjusted life years lost, years lived with disability, and years of life lost were extracted from the 2019 global burden of diseases study for regions and chartered cities in Ethiopia from 1990 to 2019. Rates were estimated per 100,000 population.

**Results:**

In 2019, the age-standardized rate of incidence was 7,118 (95% UI: 6,621–7,678), prevalence was 21,735 (95% UI: 19,251–26,302), death was 72 (95% UI: 61–83), disability-adjusted life years lost was 3,265 (95% UI: 2,826–3,783), years of live lost was 2,417 (95% UI: 2,043–2,860), and years lived with disability was 848 [95% UI: (620–1,153)]. Since 1990, there has been a reduction in the age-standardized rate of incidence by 76% (95% UI: 74–78), death by 70% (95% UI: 65–75), and prevalence by 13% (95% UI: 3–18), with noticeable inter-regional variations. Transport injuries, conflict and terrorism, interpersonal violence, self-harm, falls, poisoning, and exposure to mechanical forces were the leading causes of injury-related deaths and long-term disabilities. Since 1990, there has been a decline in the prevalence of transport injuries by 32% (95% UI: 31–33), exposure to mechanical forces by 12% (95% UI: 10–14), and interpersonal violence by 7.4% (95% UI: 5–10). However, there was an increment in falls by 8.4% (95% UI: 7–11) and conflict and terrorism by 1.5% (95% UI: 38–27).

**Conclusion:**

Even though the burden of injuries has steadily decreased at national and sub-national levels in Ethiopia over the past 30 years, it still remains to be an area of public health priority. Therefore, injury prevention and control strategies should consider regional disparities in the burden of injuries, promoting transportation safety, developing democratic culture and negotiation skills to solve disputes, using early security-interventions when conflict arises, ensuring workplace safety and improving psychological wellbeing of citizens.

## Background

Injury is a major public health problem worldwide, causing substantial premature death and serious nonfatal consequences that may lead to permanent impairment (1, 2). Injury consequences extend beyond health to have a significant macroeconomic impact. In 2019, injuries, such as road traffic crashes, unintentional injuries, self-harm, and interpersonal violence caused 4.3 million deaths globally, accounting for 10% of the total deaths and over 40 million years lived with disability (YLDs) (1, 3, 4). While injury affects all ages, youths, and young adults are particularly vulnerable (1–3, 5, 6). Moreover, the injury burden is disproportionately high in low- and middle-income countries (LMICs) (3, 4). According to the 2019 Global Burden of Diseases, Injuries, and Risk Factors (GBD) study there are significant regional variations in the global incidence of injuries (6). Over 90% of injuries that occur globally take place in LMICs, where there are inadequate safety precautions and medical services available (3, 5).

Ethiopia is a resource-constrained country where the burden of injury is unknown due to a lack of injury surveillance (7–10). Additionally, the existing studies focus on specific injury types at healthcare institutions or are small-scale surveys, which fall short of guiding evidence-based intervention strategies (11–13). However, the country has experienced frequent intergroup conflicts as a result of political disputes involving efforts to change the form of government (14–16). Unintentional injuries, such as drowning, falling, poisoning, and animal contact are growing public health problems (8, 17–20). Furthermore, road traffic injuries in particular are the leading causes of premature deaths and long-term disabilities (7, 8). For instance, a study conducted in healthcare facilities in Addis Ababa showed that road traffic injuries posed a high burden on the health facilities due to high incidence (11, 20). Approximately six out of 10 (61%) injury-related admissions and half (52%) of severe injuries have resulted in death (20). A systematic review also showed a high (32%) prevalence of road traffic injuries among trauma patients in Ethiopia, with regional disparities ranging from 14 to 60% (21).

The negative consequences of injuries to development and health have been stressed in the 2030 agenda of the sustainable development goals (SDGs) (22). Moreover, there are various goals and targets for preventing violence and injury included in the SDGs, which provide a governance framework for cross-sector preventative action (23). To determine if progress is being made, this initiative requires analyses of high-quality data. However, studies that systematically investigate injuries at national and sub-national levels are scarce in Ethiopia. The lack of such evidence impairs the design of appropriate preventive measures, and the management of survivors who need medical care and psychosocial support. Therefore, this study aimed to estimate the incidence, prevalence, mortality, disability-adjusted life years (DALYs) lost, years of life lost due to premature mortality (YLLs), and YLDs due to injuries, and the associated causes in all regional states and chartered cities in Ethiopia during 1990 to 2019 by using data from the 2019 GBD study. The findings of the study will inform policies and programs aiming at improving injury prevention strategies and societal well-being in Ethiopia.

## Methods

### Setting

Ethiopia is Africa’s second most populous country with an estimated population of 112 million in 2019 (24). The country is administratively divided into 10 regional states and two chartered cities. According to the United Nation’s world population prospect, the median age of the population is 20 years with an annual population growth rate of 2.5% in 2020 (24). Over 80% of the population resides in rural areas, where healthcare accessibility is limited (25).

### Data sources, processing, and analyses

This study used data from the 2019 GBD study accessible through GBD Compare (6, 26). The 2019 GBD study complies with the guidelines for accurate and transparent health estimates reporting recommendations (27). The causes of premature death, loss of health and prolonged disability are structured using a hierarchical classification, according to international classification of diseases (ICD) or alternative classification systems to the GBD cause list, and used for modeling to produce results that are mutually exclusive and collectively exhaustive (6). In the 2019 GBD study injury incidence and death are classified as ICD-9 codes E000-E999 and ICD-10 chapters V to Y with exception that deaths and cases of alcohol poisoning and drug overdoses are classified under drug and alcohol use disorders. A cause-specific mortality was estimated using cause of death ensemble model that produces several plausible combinations of covariates using a covariate selection algorithm (6). The study quantifies the comparative magnitude of health loss due to diseases, injuries, and risk factors stratified by age, sex, and geographies for specific points in time, and produces regular estimates of all-cause mortality, deaths by cause, DALYs, YLLs, and YLDs for a list of causes over the years 1990 to 2019 (6). The study produced these estimates for 204 countries and territories that were grouped into 21 regions and seven super-regions.

Input data for injuries are compiled from a range of sources, including international databases that capture several cause-specific fatal discontinuities and supplemental data in the presence of known issues with data quality, representativeness, or time lags in reporting (6). Based on addition of new data and change in methods, the GBD estimates are updated for the whole time series. Each metric of interest borrows strength between locations and over time through the spatiotemporal Gaussian process regression. The 2019 GBD results, thus, supersede those from previous rounds. The GBD injury-specific estimates for Ethiopia were derived from data obtained from police report, Addis Ababa mortality surveillance program, Ethiopian health and demographic surveillance system, and different surveys, like demographic and health surveys. The explicit list of data sources used in this study is available at: http://ghdx.healthdata.org/gbd-2019/data-input-sources.

In this analysis, we extracted estimates of injuries on incidence, prevalence, mortality, DALYs, YLLs, YLD, and the associated causes in all regional states and chartered cities in Ethiopia during 1990 to 2019. All rates were age-standardized and aggregated for different socio-demographic categories, regions, and cities. Trends of the metrics were also explored for the regions and charted cities over the years 1990 to 2019. The 95% uncertainty intervals (UI) were presented for each summary statistics. UIs were generated for every metric using the 25 and 97.5th ordered 1,000 draw values of the posterior distribution. The details of the estimation techniques were published elsewhere (6, 28).

## Results

### Incidence and prevalence

In 2019 there were an estimated 8.1 million (95% UI: 7.4–8.9) new cases of injury, with 4.3 million males and 3.8 million females. In the same year, there were an estimated 16.6 million (95% UI: 14.7–20.0) prevalent cases, with 8.7 million males and 7.8 million females. The age-standardized incidence rate was 7,118 cases per 100,000 population (95% UI: 6,621–7,678) and the age-standardized prevalence rate was 21,735 cases per 100,000 population (95% UI: 19,251-26,302). A higher than the national age-standardized incidence rate was observed in the Somali region [7,724 cases per 100,000 population (95% UI: 7,210–8,318)], followed by the Southern Nations and Nationalities and Peoples’ region [7,588 cases per 100,000 population (95% UI: 7,090–8,165)]. Similarly, a higher than the national age-standardized prevalence rate of injury was observed in the Gambella region [25,386 cases per 100,000 population (95% UI: 21,487–31,496)], followed by the Tigray region [24,123 cases per 100,000 population (95% UI: 20,950–29,038)]. Between 1990 and 2019, the age-standardized incidence rate was decreased by 76% (95% UI: 74–78), and the age-standardized prevalence rate by 13% (95% UI: 3–18). The highest reductions were observed in Addis Ababa, Amhara, and Benishangul-Gumuz regions (Table 1).

**Table 1:** Age-standardized rate of injury incidence, prevalence, mortality, and annualized rate of change by sex and sub-national states in Ethiopia, from 1990 to 2019.

Region	Sex	Age-standardized incidence per 100,000 population	Annualized rate of change	Age-standardized prevalence per 100,000 population	Annualized rate of change	Age-standardized mortality per 100,000 population	Annualized rate of change
		2019	1990–2019	2019	1990–2019	2019	1990–2019
All regions combined	Male	7700.2 (7167.8–8320.4)	−79.6 (−81.2, −77.7)	23800.8 (20668.5–29606.1)	−15.3 (−21.1, −4.8)	95.8 (78.6–117.3)	−71.1 (−76.5, −63.1)
	Female	6556.5 (6058.5–7,140)	−71.5 (−74.5, −67.9)	19577.1 (17583.9–22979.4)	−10.2 (−14.9, −0.8)	46.7 (39–55)	−69.5 (−75.6, −62.8)
	Both	7117.6 (6620.9–7678.4)	−76.3 (−78.4, −73.8)	21735.2 (19250.8–26302.2)	−12.7 (−18, −2.7)	71.6 (61.1–82.7)	−70.3 (−74.9, −64.6)
Tigray	Male	7512.6 (6944.3–8,152)	−77 (−79.1, −74.3)	27,007 (23116.5–33177.4)	−17.7 (−22.7, −10.2)	86.9 (65.7–113.9)	−71.4 (−78.7, −61.8)
	Female	6544.6 (6002.4–7158.4)	−63.8 (−68.4, −58.9)	21335.7 (18795.1–25338.3)	−12.6 (−16.3, −6.8)	41.9 (32.4–53.5)	−70.7 (−78, −60.8)
	Both	7002.5 (6466.1–7584.1)	−71.8 (−74.7, −68.3)	24122.8 (20949.7–29037.7)	−15 (−19.2, −8.1)	63.8 (51.8–77.9)	−71.2 (−77.1, −64.5)
Afar	Male	7963.2 (7419.9–8594.8)	−74.4 (−76.8, −71.1)	22305.2 (19607.2–27289.7)	−15.9 (−21.7, −5.8)	109.1 (79.4–147)	−66 (−76.2, −48.7)
	Female	6549.7 (6071.5–7105.4)	−67.2 (−71.2, −63.2)	18077.1 (16039–21,412)	−16.7 (−22.1, −7.1)	78.5 (61.1–101.4)	−58.1 (−70.3, −39.4)
	Both	7291.7 (6797.4–7851.9)	−71.7 (−74.6, −68.2)	20539.9 (18136.7–24795.8)	−15.5 (−21.1, −5.6)	95.4 (76.4–119)	−63.5 (−71.9, −50.9)
Amhara	Male	7493.1 (6945.1–8080.7)	−85.3 (−86.3, −84.1)	24848.2 (21461.2–30,493)	−17.1 (−22.7, −8.7)	91.4 (67.5–124.6)	−78.1 (−83.6, −71)
	Female	6165.6 (5656.4–6708.4)	−82.7 (−84.4, −80.3)	19930.9 (17608.7–23599.6)	−15.2 (−20.4, −7.5)	37.6 (29.5–47.9)	−80.8 (−84.8, −75.9)
	Both	6,820 (6316.8–7,384)	−84.1 (−85.3, −82.6)	22387.1 (19610.9–26,965)	−16.3 (−21.6, −8.2)	64.1 (51.7–81)	−79 (−83.2, −74)
Oromia	Male	7438.4 (6906.1–8049.4)	−76.7 (−78.9, −73.8)	22403.7 (19658.8–27102.5)	−18.1 (−23.7, −9.6)	81.3 (64.1–102.3)	−70.7 (−77.6, −59.3)
	Female	6,698 (6177–7305.4)	−61.5 (−66, −56.8)	20429.3 (18638.3–23200.7)	−5.6 (−10, 1.7)	45.6 (35.8–55.9)	−63.9 (−73.4, −51.5)
	Both	7060.3 (6555.2–7640.4)	−71 (−73.9, −67.4)	21458.7 (19300–25,309)	−12.1 (−17, −4)	63.8 (53.8–74.9)	−68 (−74, −58.7)
Somali	Male	8644.8 (8050.4–9314.9)	−72.7 (−75.2, −69.3)	25158.4 (22149.3–29794.7)	−1.7 (−7.4, 5.4)	103.8 (75.5–142.6)	−51.7 (−62, −38)
	Female	6683.1 (6168.4–7280.8)	−64.1 (−68.1, −59.9)	19117.4 (17035.4–22234.6)	−10.3 (−15.9, −1.6)	54.8 (40.4–72.5)	−52.3 (−65, −36.5)
	Both	7,724 (7210.1–8317.6)	−69 (−72.1, −65.4)	22640.2 (20096.4–26691.1)	−3 (−9.2, 4.9)	84.1 (66–108.2)	−59.8 (−70.5, −45.2)
Benishangul-Gumuz	Male	7794.8 (7259–8,404)	−78.2 (−80, −75.8)	22644.4 (19778.6–27873.1)	−15.9 (−21.9, −5.5)	95.9 (71.7–129.2)	−67.5 (−74.3, −57.7)
	Female	6505.7 (6057.2–7045.3)	−69.1 (−72.9, −65.1)	18275.7 (16453.4–21254.8)	−16.3 (−21, −7.4)	70.3 (53.2–88.8)	−69.5 (−77.9, −56.2)
	Both	7148.8 (6691.6–7,702)	−74.6 (−77, −71.7)	20621.4 (18307.9–24,830)	−15.8 (−21.4, −6.2)	83.9 (67.6–103.6)	−64.6 (−74.9, −50.4)
Southern Nations, Nationalities	Male	8271.6 (7700.6–8920.7)	−74.1 (−76.5, −70.9)	23584.6 (20284.4–32674.6)	−11.2 (−18.9, 12.7)	135 (104.3–167.1)	−60 (−69.8, −46)
	Female	6949.1 (6442.6–7521.3)	−60.5 (−65.1, −55.5)	17,835 (15805.2–22725.6)	−8.1 (−14.2, 12.1)	66 (51.3–81)	−54 (−66.3, −39.1)
	Both	7588.1 (7090.1–8165.1)	−68.8 (−72, −65.1)	20726.6 (18113.4–27915.8)	−9.6 (−16.6, 13.4)	100.6 (81–119)	−58.3 (−66.7, −48.3)
Gambella	Male	7608.8 (7064.8–8220.8)	−77.4 (−79.4, −74.5)	29316.2 (24337.9–37178.9)	−3.6 (−11.6, 5)	117.2 (89.3–150.5)	−62.1 (−70, −50.5)
	Female	6068.3 (5594.3–6,609)	−62.3 (−66.5, −57.8)	22047.8 (18926.8–27039.1)	7.2 (−3.2, 23.4)	33.2 (26.3–40.5)	−64.7 (−74.7, −49.3)
	Both	6787.4 (6304.7–7330.8)	−71 (−73.9, −67.7)	25386.4 (21486.9–31495.5)	3.1 (−6, 14.5)	73.8 (60.8–89.9)	−66.6 (−76.6, −54.1)
Harari	Male	6906.1 (6380–7479.1)	−80.8 (−82.5, −78.5)	25238.6 (21631.5–31019.3)	−14.4 (−23, −3.3)	86.9 (63.5–120.9)	−71.1 (−78.3, −62.1)
	Female	6072.8 (5604.7–6600.3)	−64.9 (−69.1, −60.6)	20167.2 (17508.8–24360.7)	1.3 (−8.3, 16.6)	40.3 (30.3–53.1)	−89.1 (−92.1, −84.8)
	Both	6504.7 (6031.2–7054.6)	−72.7 (−75.6, −69.1)	22673.4 (19609.9–27456.6)	−1.3 (−10.6, 11.4)	62.6 (49.8–79.2)	−68 (−77.7, −53.9)
Dire Dawa	Male	7218.6 (6677.5–7813.5)	−76.4 (−78.7, −73.4)	23231.4 (20271.7–28388.4)	−13 (−19.6, −3)	81.1 (59.9–113.6)	−70.7 (−78.9, −56.6)
	Female	6119.8 (5641.3–6671.8)	−65.2 (−69.3, −60.8)	18455.7 (16445–21692.7)	−7 (−13.2, 3.4)	37.5 (28.3–49.5)	−67.7 (−77.5, −53.2)
	Both	6664.3 (6173.3–7212.6)	−72 (−75, −68.5)	20802.2 (18407.1–25,010)	−9.7 (−16, 0.5)	58.7 (46–74.2)	−68.8 (−75.6, −59.3)
Addis Ababa	Male	7191.4 (6646.4–7,820)	−86 (−87, −84.9)	23367.9 (20207.8–28719.1)	−19.4 (−25.9, −8.4)	76.9 (58.7–98.3)	−80.8 (−84, −76.8)
	Female	6058.7 (5524.1–6649.5)	−81.9 (−83.8, −79.6)	18234.3 (16036.1–21771.2)	−19 (−24.9, −10.1)	32 (25–43.3)	−79.2 (−84.2, −73.2)
	Both	6568.9 (6053–7152.7)	−84.4 (−85.6, −82.9)	20713.2 (18131.2–25180.7)	−19.4 (−25.5, −9)	52.8 (44.5–63.8)	−83.1 (−87, −76.8)

### Mortality

**Figure 1:**
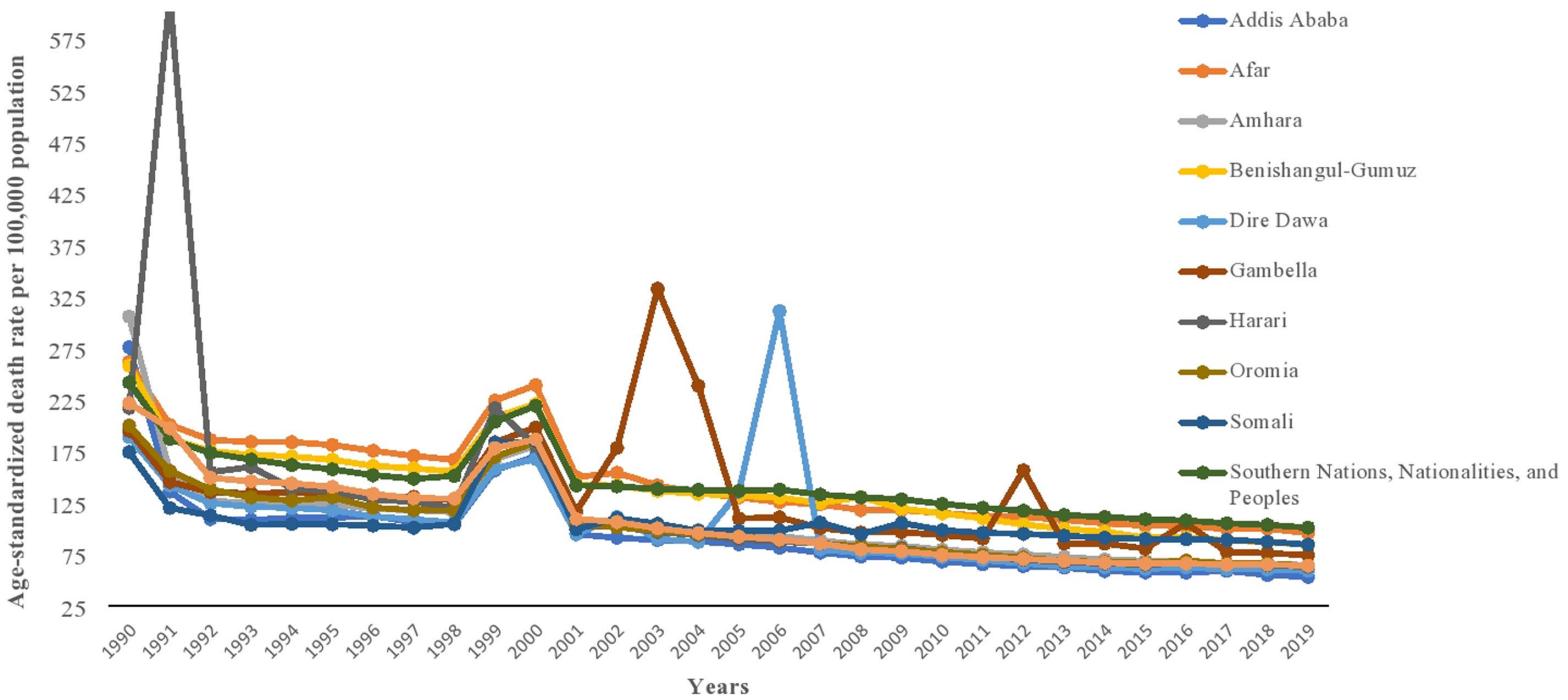
Trend of age-standardized death rate due to injury in Ethiopia, 1990–2019.

An estimated 43,658 (95% UI: 37,027-51,499) injury-related deaths occurred in 2019, with 30,430 deaths among males and 13,228 deaths among females. This accounted for about 7.8% (9.5% males vs. 5.4% females) of deaths from all-causes in Ethiopia. The corresponding age-standardized death rate was estimated to be 72 deaths per 100,000 population (95% UI: 61–83), with 96 deaths per 100,000 males and 47 deaths per 100,000 females. A higher than the national age-standardized death rate of injury was occurred in the Southern Nations Nationalities and Peoples’ region [101 deaths per 100,000 population (95% UI: 81–119)], Afar region [95 deaths per 100,000 population (95% UI: 76–119)], Somali region [84 deaths per 100,000 population (95% UI: 66–108)], Benishangul-Gumuz region [84 deaths per 100,000 population (95% UI: 68–104)], and Gambella region [74 deaths per 100,000 population (95% UI: 61–90)]. There was a 70% (95% UI: 65–75) reduction in injury-related death rate between 1990 and 2019, with noticeable inter-regional disparities (Table 1; Figure 1).

### Disability-adjusted life years lost

**Figure 2:**
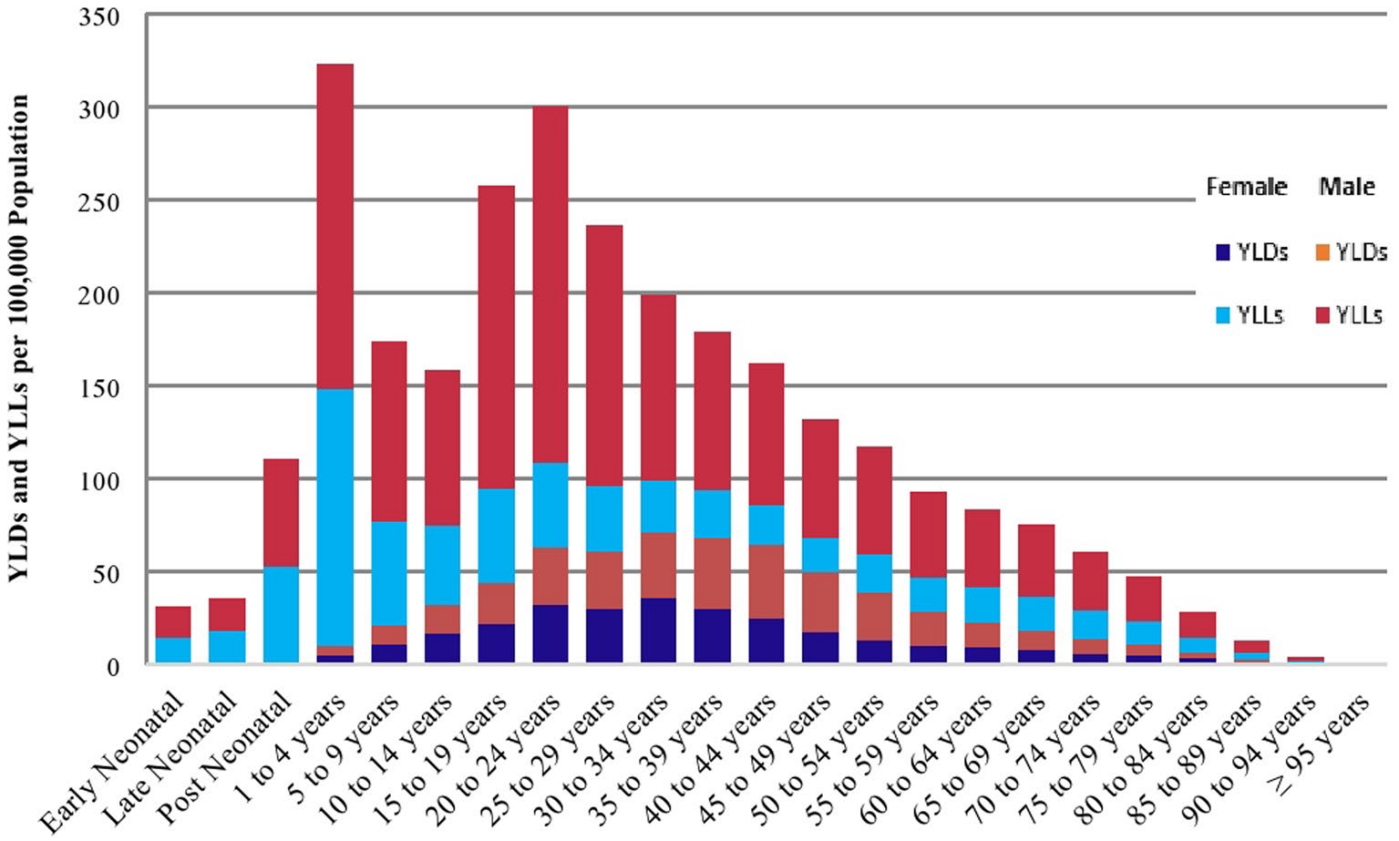
YLDs and YLLs by age and sex in Ethiopia, 2019.

Injuries resulted an estimated 2.8 million (95% UI: 2.4–3.0) DALYs lost in 2019, which accounted for about 7.4% (9.0% males vs. 6.4% females) of the disability-adjusted life years (DALYs) lost from all-causes in Ethiopia. The corresponding age-standardized rate of DALYs lost was estimated to be 3,265 years per 100,000 population (95% UI: 2,826–3,783), which was more than three-times lower than the 1990 estimate of 11,386 years per 100,000 population (95% UI: 10,561–12,165). A higher than the national age-standardized rate of DALYs lost was observed in the Southern Nations Nationalities and Peoples’ region [4,227 years per 100,000 population (95% UI: 3,482–5,083)], Afar region [4,041 years per 100,000 population (95% UI: 3,307-4,975)], Benishangul-Gumuz region [3,950 years per 100,000 population (95% UI: 3,299–4,716)], Somali region [3,884 years per 100,000 population (95% UI: 3,174–4,797)], and Gambella region [3,362 years per 100,000 population (95% UI: 2,751–4,070); Table 2]. The burden of DALYs lost due to injuries increased until the age of 24 years and then decreased as age increased, (Figure 2).

**Table 2:** Age-standardized rate of injury-related DALYs, YLLs, YLDs, and annualized rate of change by sex and sub-national states in Ethiopia, from 1990 to 2019.

Region	Sex	Age-standardized DALYs per 100,000 population	Annualized rate of change	Age-standardized YLLs per 100,000 population	Annualized rate of change	Age-standardized YLDs per 100,000 population	Annualized rate of change
		2019	1990–2019	2019	1990–2019	2019	1990–2019
All regions combined	Male	4320.1 (3642.3–5133.5)	−74.7 (−78.8, −69)	3359.8 (2752.8–4112.3)	−78.5 (−82.6, −72.8)	960.3 (696.3–1323.3)	−33.2 (−39.9, −20.7)
	Female	2196.6 (1859.5–2,603)	−73.6 (−77.7, −68.5)	1463.7 (1188.3–1794.5)	−80 (−84.1, −75.1)	732.9 (544.3–981.6)	−26.5 (−33.1, −15.9)
	Both	3264.9 (2825.5–3782.9)	−74.1 (−77.5, −69.7)	2416.9 (2042.8–2,860)	−78.8 (−82.2, −74.3)	848 (620.1–1153.2)	−29.9 (−36.3, −18.4)
Tigray	Male	3994.5 (3214.2–4986.8)	−73.7 (−79, −67.1)	2888.8 (2165–3791.8)	−78.3 (−83.9, −71.1)	1105.7 (807.9–1486.3)	−39.4 (−45.9, −31.4)
	Female	1984.5 (1599.3–2438.7)	−72.9 (−78.1, −66.9)	1172.1 (877.2–1,569)	−81 (−86.3, −74.3)	812.4 (599.9–1077.2)	−29.4 (−35.2, −23.1)
	Both	2965.5 (2479.1–3534.6)	−73.2 (−77.6, −67.9)	2009.4 (1587.7–2530.2)	−79.1 (−83.5, −73.7)	956.2 (702.5–1281.6)	−34.7 (−40.6, −27.4)
Afar	Male	4,851 (3681.1–6384.9)	−68.5 (−77.1, −55.3)	3945.1 (2815.8–5428.8)	−72.2 (−80.9, −58.5)	905.9 (658.6–1,237)	−26.4 (−32.6, −15.6)
	Female	3,100 (2461.5–3,901)	−66.7 (−75, −55.4)	2405.4 (1808.8–3161.8)	−71.4 (−79.6, −59.1)	694.6 (512–922.8)	−22.8 (−28.4, −14.5)
	Both	4041.1 (3307.2–4974.6)	−68 (−74.8, −59)	3233.8 (2488.6–4128.7)	−72 (−78.9, −62.8)	807.3 (588.9–1095.2)	−24.8 (−30.7, −14.7)
Amhara	Male	4202.7 (3326.2–5447.7)	−81.8 (−85.3, −77)	3203.2 (2408.9–4408.4)	−85 (−88.7, −80.1)	999.6 (730.8–1,352)	−39.7 (−46.4, −31.1)
	Female	1939.9 (1593.1–2362.8)	−83.1 (−85.9, −79.4)	1192.7 (924.8–1547.1)	−88.4 (−91, −84.8)	747.2 (554.6–989.7)	−36 (−43.6, −28.3)
	Both	3066.8 (2555.2–3751.3)	−82.1 (−84.9, −78.6)	2193.8 (1744.6–2826.7)	−86 (−88.8, −82.6)	873.1 (641.6–1170.6)	−37.9 (−44.6, −29.8)
Oromia	Male	3648.8 (3052.4–4,394)	−73.4 (−78.5, −65.6)	2747.9 (2200.3–3445.8)	−77.9 (−82.9, −69.2)	900.8 (657.2–1228.8)	−31.6 (−37.5, −22.3)
	Female	2083.4 (1722.6–2539.6)	−68.4 (−74.7, −60)	1356.9 (1040.2–1757.3)	−76.2 (−82.6, −67.8)	726.5 (542.9–953.8)	−20 (−25.7, −13)
	Both	2874.7 (2468.6–3349.1)	−71.4 (−75.8, −64.9)	2059.4 (1715.9–2,480)	−77 (−81.5, −70.2)	815.3 (598.8–1098.2)	−26.2 (−31.8, −18.1)
Somali	Male	4881.3 (3805.6–6,351)	−55.7 (−64.5, −45.1)	3842.8 (2833–5239.4)	−60.5 (−70.5, −48.8)	1038.5 (761.6–1390.9)	−19.1 (−25.3, −12.5)
	Female	2613.7 (2057–3272.5)	−61.7 (−69.7, −51.9)	1856.5 (1333.3–2486.8)	−68.5 (−77, −57.9)	757.1 (564.6–987.1)	−17.8 (−23.5, −11)
	Both	3883.9 (3174.4–4797.4)	−56.1 (−63.5, −48)	2,970 (2328.6–3767.6)	−61.7 (−69.7, −52.9)	913.9 (668.7–1212.8)	−16.7 (−22.7, −10)
Benishangul-Gumuz	Male	4662.7 (3672.1–5906.1)	−71.7 (−77.7, −62.9)	3734.8 (2816.1–4937.4)	−75.4 (−81.7, −66)	927.9 (669.2–1,273)	−29.3 (−35.8, −18.1)
	Female	3194.3 (2508.3–3915.8)	−69.2 (−76.6, −59.8)	2498.3 (1867–3,195)	−73.6 (−81.1, −64.1)	695.9 (516.7–917.4)	−23.6 (−29.2, −15.3)
	Both	3950.2 (3299.4–4716.3)	−70.5 (−75.7, −64)	3131.8 (2507.3–3895.7)	−74.5 (−80.1, −68)	818.3 (598.9–1105.1)	−26.4 (−32.3, −16.6)
Southern Nations, Nationalities	Male	5708.2 (4541.8–7053.8)	−65.2 (−72.5, −55.1)	4754.9 (3664.9–5950.2)	−68.5 (−76.1, −57.9)	953.3 (651.9–1508.2)	−26.2 (−36.2, 6.2)
	Female	2,759 (2173–3437.5)	−62.8 (−71.6, −52.2)	2043.5 (1508.8–2636.8)	−68.7 (−77.8, −57.7)	715.5 (509.8–1,061)	−20.2 (−28.7, 4)
	Both	4226.5 (3481.8–5082.8)	−64.4 (−71, −56.4)	3391.9 (2704.5–4101.4)	−68.5 (−75.2, −60.7)	834.6 (581.7–1285.4)	−23.3 (−32.5, 6.1)
Gambella	Male	5110.2 (4022.8–6468.1)	−69.6 (−77.1, −58.7)	3910.1 (2888.2–5165.5)	−74.4 (−82, −63.2)	1200.1 (866.9–1623.2)	−21.7 (−27.8, −15)
	Female	1749 (1434.1–2126.9)	−71.6 (−77.9, −63.9)	873.8 (678.1–1107.3)	−83.3 (−88.2, −77.1)	875.2 (637.4–1182.1)	−6.6 (−16.5, 6.7)
	Both	3361.7 (2751.1–4069.8)	−68.5 (−74.6, −60.5)	2338.6 (1849.7–2945.7)	−75.4 (−81.2, −67.2)	1,023 (740.7–1390.6)	−13.7 (−21.1, −4.7)
Harari	Male	4048.5 (3181.7–5210.9)	−83.6 (−87.4, −78.3)	2944.2 (2120.2–4,105)	−87.3 (−90.9, −81.9)	1104.3 (802.7–1488.9)	−28.6 (−34.7, −21.1)
	Female	2033.4 (1575.2–2607.8)	−71.2 (−78.2, −62.4)	1170.8 (832.4–1657.3)	−80.9 (−86.9, −72.1)	862.5 (628.4–1165.4)	−7 (−17.4, 7.8)
	Both	3036.4 (2471.9–3712.5)	−73.7 (−78.9, −66.7)	2050.8 (1569.1–2,677)	−80.3 (−85.2, −73.4)	985.6 (715–1325.2)	−11.1 (−20.7, 1.8)
Dire Dawa	Male	3693.5 (2885.3–4,816)	−73.8 (−79.9, −64.3)	2731.1 (2003.6–3,820)	−78.6 (−84.9, −69)	962.4 (706.2–1303.2)	−26.6 (−33.3, −17.2)
	Female	1807.4 (1423.8–2,290)	−73.5 (−79.4, −65.4)	1068.4 (761.5–1534.4)	−81.9 (−87.7, −72.9)	739 (552–974.4)	−20.5 (−27, −11.6)
	Both	2737.2 (2213.8–3358.6)	−73.5 (−78.8, −66.9)	1887.4 (1459.4–2456.3)	−79.5 (−84.5, −72.6)	849.8 (628.5–1134.2)	−23.7 (−30.1, −14.6)
Addis Ababa	Male	3491.7 (2788–4382.7)	−82.4 (−86, −78)	2590.3 (1938.5–3403.4)	−85.8 (−89.4, −81.2)	901.4 (659.2–1236.2)	−43.1 (−49.8, −33.4)
	Female	1,560 (1242.3–1981.8)	−85 (−87.9, −81)	887.8 (640.1–1246.9)	−90.4 (−93.1, −86.3)	672.2 (494.5–900.6)	−40.9 (−47.6, −33.2)
	Both	2457.5 (2033.3–2,952)	−83.5 (−86.2, −80.1)	1,673 (1321.3–2124.3)	−87.6 (−90.3, −84.3)	784.4 (574.2–1061.2)	−42.1 (−48.7, −33.3)

### Years of life lost

There were an estimated 2.2 million (95% UI: 1.8–2.6) years of life lost (YLLs) in 2019, which was nearly three-times lower than the 1990 estimate of 6.1 million (95% UI: 6.1–6.5) YLLs in Ethiopia. The corresponding age-standardized rate of YLLs as a result of injuries was 2,417 years per 100,000 population (95% UI: 2,043–2,860). A higher than the national age-standardized rate of YLLs was observed in the Southern Nations Nationalities and Peoples’ region [3,392 years per 100,000 population (95% UI: 2,705–4,101)], Afar region [3,234 years per 100,000 population (95% UI: 2,489–4,129)], Benishangul-Gumuz region [3,132 years per 100,000 population (95% UI: 2,507–3,896)], and Somali region [2,970 per 100,000 population (95% UI: 2,329–3,768)]. Males encountered a higher rate of YLLs in each region across the country (Table 2). The burden of YLLs due to injuries increased until the age of 24 years and then decreased as age increased (Figure 2).

### Years lived with disability

In 2019 injuries contributed to 630,454 (95% UI: 460,443–865,634) YLDs in Ethiopia, with the corresponding age-standardized rate of 848 years per 100,000 population [95% UI: (620–1,153)]. A higher than the national age-standardized rate of YLDs was observed in Gambella region [1,023 years per 100,000 population (95% UI: 741–1,391)], Harari region [986 years per 100,000 population (95% UI: 715–1,325)], Tigray region [956 years per 100,000 population (95% UI: 703–1,282)], Somali region [914 years per 100,000 population (95% UI: 669–1,213)], Amhara region [873 years per 100,000 population (95% UI: 642–1,171)], and Dire Dawa city administration [850 years per 100,000 population (95% UI: 629–1,134)]. The rate of YLDs was higher among males in each region across the country (Table 2). The burden of YLDs due to injuries increased until the age of 34 years and then decreased as age increased (Figure 2).

### The leading causes of injuries

The top-five leading causes of incident injuries in 2019 were exposure to mechanical forces [1,381 cases per 100,000 population (95% UI: 1,098–1,674)], animal contacts [1,209 cases per 100,000 population (95% UI: 1,000–1,486)], falls [1,162 cases per 100,000 population (95% UI: 958–1,400)], foreign bodies [1,158 cases per 100,000 population (95% UI: 939–1,434)], and transport injuries [1,089 cases per 100,000 population (95% UI: 927–1,277)]. Similarly, the most common causes of prevalent injuries were conflict and terrorism [4,889 cases per 100,000 population (95% UI: 2,608–9,342)], interpersonal violence [3,640 cases per 100,000 population (95% UI: 3,208–4,175)], transport injuries [3,183 cases per 100,000 population (95% UI: 2,962–3,451)], exposure to mechanical forces [2,688 cases per 100,000 population (95% UI: 2,327–3,060)], and falls [2,440 cases per 100,000 population (95% UI: 2,116–2,823)]. Since 1990, there has been a decline in the prevalence of transport injuries by 32% (95% UI: 31–33), motorcycle injuries by 29% (95% UI: 27–31), motor vehicle injuries by 28% (95% UI: 26–30), exposure to mechanical forces by 12% (95% UI: 10–14), and interpersonal violence by 7.4% (95% UI: 5–10). However, there was an increment in the prevalence of falls by 8.4% (95% UI: 7–11) and conflict and terrorism by 1.5% (95% UI: 38–27; Table 3).

**Table 3:** Age-standardized rate of incidence, prevalence and mortality by injury cause and annualized rate of change in Ethiopia, from 1990 to 2019.

Causes of injury	Age-standardized incidence per 100,000 population	Annualized rate of change	Age-standardized prevalence per 100,000 population	Annualized rate of change	Age-standardized mortality per 100,000 population	Annualized rate of change
	2019	1990–2019	2019	1990–2019	2019	1990–2019
Transport injuries	1,089 (926.5–1276.5)	−31.3 (−33, −29.5)	3183.2 (2962.4–3451.1)	−32 (−32.9, −31.1)	15.2 (12.8–18.2)	−54.6 (−66.2, −40.4)
Road	1044.7 (884.1–1233.5)	−31.8 (−33.6, −30)	2983.6 (2759.1–3238.2)	−32.8 (−33.7, −31.9)	14.5 (12.2–17.4)	−54.3 (−65.8, −39.8)
Pedestrian	298.3 (237.1–371.6)	−41.5 (−43.8, −39)	1072.4 (935.8–1234.6)	−42.1 (−43.3, −40.8)	6.3 (4.7–8.4)	−60.7 (−72.8, −39.4)
Cyclist	403.6 (320.2–508.6)	−26.8 (−29.4, −23.9)	921.2 (780.3–1108.3)	−24.8 (−26, −22.9)	0.7 (0.4–1.1)	−54.8 (−75.6, −30.9)
Motor vehicle	214.6 (161.7–269.8)	−30.1 (−33.1, −26.8)	573.1 (490–673.3)	−28 (−29.5, −26.4)	6.4 (4.9–7.9)	−45.8 (−61.4, −19.4)
Motorcyclist	97.1 (78.4–120.1)	−25.8 (−29.4, −21.7)	361 (315.3–410.7)	−28.8 (−30.5, −26.6)	1.1 (0.6–1.8)	−52.3 (−76.7, −17.4)
Other road injuries	31.2 (20.4–44.6)	−5.4 (−10.9, 0.1)	56 (47.5–64.7)	−4.9 (−7.3, −2.7)	0.1 (0–0.1)	−60.7 (−81.9, −29.5)
Other transport injuries	44.3 (34.4–56.8)	−17.4 (−24.2, −10.9)	199.6 (163.1–239.5)	−18.5 (−21.1, −15.3)	0.7 (0.5–1.2)	−61.1 (−75.6, −42.9)
Falls	1162.1 (958.4–1400.2)	9 (5.3, 12.9)	2440.3 (2116–2822.5)	8.4 (6.9, 10.5)	9.6 (8.1–11.3)	−21.3 (−40.5, 12.5)
Drowning	5.4 (4.3–6.7)	−28.1 (−33.7, −22.6)	20.9 (18.7–23.5)	−29.4 (−31.7, −27.4)	1.8 (1.5–2.6)	−67.5 (−75.3, −48.6)
Environmental heat and cold exposure	66.9 (49.3–89.3)	−8.3 (−14.2, −3)	234.6 (185–292.4)	−6.9 (−10.2, −4.3)	1.2 (0.7–1.7)	−54 (−67.4, −36.6)
Fire, heat, and hot substances	172.4 (128.5–219.7)	−23.2 (−29, −17.9)	1749 (1465.5–2,110)	−23.9 (−26.6, −21.1)	3.4 (2.4–4.8)	−47.9 (−63.7, −8.5)
Poisonings	26.8 (20.1–34.9)	−35.7 (−40.7, −30.1)	26.6 (22.8–31.2)	−38.6 (−41.8, −35.7)	4.1 (2.5–5.1)	−61.4 (−70.8, −43.7)
Poisoning by other means	20.3 (14.7–27)	−41.3 (−46.4, −36)	20 (16.4–24.5)	−44.8 (−47.5, −42)	2.1 (1.1–2.9)	−73.9 (−81.6, −58.6)
Poisoning by carbon monoxide	6.5 (4.3–9.7)	−8.1 (−17.8, 2.1)	6.5 (5–8.7)	−6.1 (−10.5, −2.4)	2 (1.3–2.7)	−22.9 (−54.3, 26.9)
Exposure to mechanical forces	1381.1 (1098–1674.3)	−11.7 (−15.8, −7.8)	2687.8 (2326.9–3060.1)	−11.7 (−13.7, −9.7)	4.4 (3.2–5.7)	−41.2 (−59.8, −16.3)
Adverse effects of medical treatment	110.3 (90.5–133)	−26 (−29.4, −22.4)	8.4 (6.2–10.9)	−26 (−29.3, −22.4)	3 (1.5–7.8)	−58.9 (−69.5, −44.9)
Animal contact	1208.7 (1000–1485.5)	−31.3 (−33.9, −28.5)	696.3 (595.3–811.2)	−31.4 (−32.7, −30.2)	3.6 (2.8–5.1)	−53.8 (−68, −19.8)
Non-venomous animal contact	852.6 (691.5–1072.3)	−31.2 (−33.9, −28.6)	551.9 (460.8–665.4)	−32.2 (−33.8, −30.7)	1.4 (0.9–2.7)	−54.5 (−76.1, 6.2)
Foreign body	1157.7 (939–1434.1)	1 (−0.8, 3.2)	808.4 (667.8–954.8)	−0.6 (−2.5, 1.4)	2.4 (2.1–2.8)	−41 (−52.9, −26.2)
Other exposure to mechanical forces	1340.4 (1065–1631.4)	−10.9 (−14.9, −7)	2564.8 (2202.3–2943.1)	−10.5 (−12.6, −8.5)	2.5 (1.6–3.6)	−25.9 (−53.4, 14.8)
Unintentional firearm injuries	40.7 (29.9–54.4)	−33.1 (−38.2, −26.4)	123 (106.6–144.1)	−30.4 (−33.6, −27.3)	1.8 (1.2–2.5)	−54.3 (−73.2, −31.6)
Pulmonary aspiration and foreign body in airway	19.5 (13.3–30)	−26 (−33.2, −18)	34.7 (26.2–46.5)	−25.8 (−30.4, −21.3)	1.6 (1.2–2.1)	−33.7 (−49.3, −12.1)
Venomous animal contacts	356.1 (261.2–475.2)	−31.4 (−34.5, −28)	144.3 (116.1–181.2)	−27.9 (−30, −25.8)	2.2 (1.6–2.9)	−53.4 (−68.1, −30.5)
Self-harm	26.9 (22.6–32.3)	−40.7 (−42.6, −38.5)	92.9 (79.3–105.1)	−41.8 (−43.6, −39.6)	10.1 (8.2–12.9)	−53.3 (−65.3, −32.7)
Interpersonal violence	231.1 (186.8–274.4)	−27.1 (−29.3, −24.5)	3639.8 (3208–4,175)	−7.4 (−9.8, −5)	9.7 (7.9–11.9)	−37.9 (−57, −13.3)
Conflict and terrorism	39.3 (30.9–48.2)	−99.8 (−99.8, −99.8)	4889.4 (2608.3–9341.9)	1.5 (−26.7, 37.7)	0.2 (0.1–0.2)	−99.8 (−99.9, −99.8)
Physical violence by firearm	3.8 (3–5.1)	−21 (−25.7, −15.6)	11.1 (9.6–12.6)	−26.9 (−28.8, −24.9)	2.9 (2.2–3.7)	−37.7 (−63.9, −4.9)
Executions and police conflict	10.9 (7.8–14.3)	0 (0, 0)	87.6 (52.8–138.3)	0 (0, 0)	0.1 (0.1–0.1)	934.3 (705.2, 1166.4)
Physical violence by other means	149.8 (119.3–184.2)	−29.1 (−31.5, −26.5)	250.7 (215.4–297.2)	−30.3 (−32.1, −28.8)	3.8 (2.9–4.7)	−39.7 (−61.6, −12.7)
Physical violence by sharp object	77.5 (60.3–95.5)	−23.1 (−26.1, −19.8)	169.7 (149.5–194.5)	−26.5 (−28, −25)	3 (2.3–3.8)	−35.6 (−58, 1.6)
Self-harm by firearm	2.2 (1.5–3.2)	−26.7 (−32, −20.4)	6.8 (5.8–8)	−24 (−27.1, −20.6)	0.5 (0.3–0.9)	−50.2 (−74.9, −11.8)
Self-harm by other specified means	1162.1 (958.4–1400.2)	−41.7 (−43.6, −39.6)	86.1 (72.4–98.7)	−42.8 (−44.7, −40.6)	9.6 (7.8–12.4)	−53.4 (−65.6, −33.4)

The top-five leading causes of injury-related deaths in 2019 were transport injuries [15 deaths per 100,000 population (95% UI: 13–18)], interpersonal violence [10 deaths per 100,000 population (95% UI: 8–12)], self-harm [10 deaths per 100,000 population (95% UI: 8–13)], falls [10 deaths per 100,000 population (95% UI: 8–11)], and poisoning [four deaths per 100,000 population (95% UI: 3–5)]. Since 1990, there was a noticeable reduction in the death rate due to poisoning by 61% (95% UI: 44–71), transport injuries by 55% (95% UI: 40–66), self-harm by 53% (95% UI: 33–65), interpersonal violence by 38% (95% UI: 13–57), and falls by 21% (95% UI: 13–41; Table 3).

The top-five leading causes of DALYs lost in 2019 were transport injuries [760 years per 100,000 population (95% UI: 647–891)], interpersonal violence [457 years per 100,000 population (95% UI: 381–557)], self-harm [333 years per 100,000 population (95% UI: 268–419)], falls [300 years per 100,000 population (95% UI: 252–354)], and exposure to mechanical forces [221 years per 100,000 population (95% UI: 166–285)]. Since 1990, there was a remarkable reduction in DALYs lost due to self-harm by 57% (95% UI: 39–69), transport injuries by 56% (95% UI: 44–66), interpersonal violence by 39% (95% UI: 16–56), exposure to mechanical forces by 38% (95% UI: 16–57), and falls by 23% (95% UI: 2–39). Furthermore, the major causes of YLDs were transport injuries [216 years per 100,000 population (95% UI: 159–283)], conflict and terrorism [212 years per 100,000 population (95% UI: 116–431)], and falls [123 years per 100,000 population (95% UI: 89–166)]. The leading causes of YLLs due to injuries were transport injuries [545 years per 100,000 population (95% UI: 452–661)], interpersonal violence [408 years per 100,000 population (95% UI: 333–505)], and self-harm [325 years per 100,000 population (95% UI: 259–411); Table 4].

**Table 4:** Age-standardized rate of DALYs, YLDs, and YLLs by injury cause and annualized rate of change in Ethiopia, from 1990 to 2019.

Causes of injury	Age-standardized DALYs per 100,000 population	Annualized rate of change	Age-standardized YLDs per 100,000 population	Annualized rate of change	Age-standardized YLLs per 100,000 population	Annualized rate of change
	2019	1990–2019	2019	1990–2019	2019	1990–2019
Transport injuries	760.4 (646.6–891.3)	−56 (−65.9, −44.1)	215.6 (158.5–283)	−32.1 (−33.3, −31.1)	544.9 (452.2–660.8)	−61.4 (−71.8, −47.4)
Road	712.4 (604.4–836.7)	−55.9 (−65.7, −44.1)	201.7 (148–265.3)	−32.7 (−33.8, −31.6)	510.7 (422.7–615.9)	−61.2 (−71.8, −47.2)
Pedestrian	290.3 (231.9–366.2)	−63.4 (−73.3, −47.2)	72.2 (50.6–96.6)	−42.7 (−44.1, −41.1)	218 (162.6–295.4)	−67.3 (−78, −48.2)
Cyclist	81.9 (61.9–103.6)	−40.6 (−55.2, −29.1)	59.8 (42.8–79.8)	−23.6 (−25.3, −21.8)	22.2 (13.3–36.4)	−62.8 (−81.2, −39.5)
Motor vehicle	269.6 (212.8–330.8)	−49.8 (−63.4, −24.4)	42 (30.3–56.2)	−26.9 (−28.7, −25.1)	227.6 (172.3–289.9)	−52.5 (−66.9, −24)
Motorcyclist	64.8 (43.3–90.2)	−52.2 (−71.4, −26.7)	24.5 (17.7–32.8)	−29.1 (−30.9, −27.1)	40.2 (22–66.7)	−60.1 (−81.6, −25.5)
Other road injuries	5.8 (4.5–7.3)	−47.3 (−69.3, −16.8)	3.1 (2.2–4.2)	−3 (−5.4, −0.8)	2.7 (1.8–3.8)	−65.4 (−85.2, −29.7)
Other transport injuries	48.1 (35.4–69.5)	−57.5 (−70.5, −42.5)	13.8 (9.9–18.2)	−22.7 (−25, −20.2)	34.2 (22.8–55)	−64.1 (−77.5, −47.2)
Falls	299.5 (251.9–354.4)	−23.1 (−38.6, 2.4)	122.5 (88.6–165.7)	7.1 (5.3, 8.9)	177 (147.3–213)	−35.7 (−53.7, −0.1)
Drowning	93.6 (72.6–145)	−73.5 (−80.9, −54.9)	1.4 (1–1.8)	−32.2 (−35.1, −29.4)	92.2 (71.3–143.6)	−73.8 (−81.1, −55)
Environmental heat and cold exposure	48.6 (32.2–67.9)	−52.7 (−68, −34.9)	10.8 (7.6–14.6)	−10.7 (−13.9, −8.1)	37.7 (22.7–56.6)	−58.3 (−73.8, −38.7)
Fire, heat, and hot substances	186.7 (142.8–245.3)	−50.7 (−64.3, −19)	72.9 (52.8–97.4)	−29.7 (−33.3, −26.1)	113.8 (76.6–167.3)	−58.7 (−73.9, −12.9)
Poisonings	151.1 (93.1–194.6)	−68.3 (−76.6, −48.5)	3 (2–4.2)	−39.4 (−43.9, −34.7)	148.1 (90–191.2)	−68.6 (−76.9, −48.7)
Poisoning by other means	91.7 (51.3–128.6)	−76.8 (−83.9, −59.5)	2.3 (1.4–3.2)	−45.9 (−49.8, −41.5)	89.4 (48.8–126.2)	−77.1 (−84.3, −59.6)
Poisoning by carbon monoxide	59.5 (37.8–86)	−27.6 (−60.1, 28)	0.8 (0.5–1.1)	−5.9 (−12.9, 0.8)	58.7 (36.9–85.3)	−27.8 (−60.4, 28.5)
Exposure to mechanical forces	221 (165.8–285.4)	−37.8 (−57, −15.5)	64.8 (45–91.1)	−14.8 (−16.9, −12.7)	156.2 (103.4–216.8)	−44 (−66.4, −15.9)
Adverse effects of medical treatment	94.9 (52.3–218.2)	−68.1 (−79.8, −50.5)	1.1 (0.7–1.7)	−26 (−29.3, −22.4)	93.8 (51–217.3)	−68.3 (−80, −50.6)
Animal contact	132.2 (100.6–188.4)	−56.4 (−70, −23.4)	14.8 (10–21.1)	−33.5 (−35.6, −31.7)	117.5 (86.7–173.6)	−58.2 (−72.1, −22.3)
Non-venomous animal contact	61.4 (42.9–101)	−59.2 (−76.1, −12)	9.4 (6.2–13.9)	−36.7 (−39.6, −34.5)	52 (34.6–93.3)	−61.7 (−79.4, −8.1)
Foreign body	116.3 (99.2–133.5)	−41.1 (−52.2, −27.6)	32.3 (22.9–44.2)	−3.7 (−5.9, −1.6)	83.9 (70.5–99.6)	−48.8 (−60.6, −33.5)
Other exposure to mechanical forces	154.9 (107.8–207.4)	−24.9 (−48.7, 7.1)	57.5 (39.7–81.4)	−12.5 (−14.6, −10.5)	97.4 (57.3–148.9)	−30.7 (−60, 17.3)
Unintentional firearm injuries	66 (42.9–89.7)	−55.6 (−74, −32.7)	7.3 (5.2–9.6)	−29.6 (−32.4, −26.1)	58.8 (36.3–81.7)	−57.5 (−76.7, −33.1)
Pulmonary aspiration and foreign body in airway	56.4 (41.3–73.1)	−41.6 (−57.2, −19.1)	2.1 (1.5–2.9)	−29 (−33.2, −24.8)	54.2 (39.1–70.9)	−42 (−58, −18.8)
Venomous animal contacts	70.9 (53–97.2)	−53.7 (−67.8, −28.9)	5.4 (3.6–7.4)	−26.9 (−30.1, −23.8)	65.5 (47.6–91.4)	−55 (−69.8, −28.9)
Self-harm	333.1 (268–419)	−57.4 (−69, −39)	8.4 (6.1–11.1)	−45.6 (−48, −43)	324.7 (259.3–411)	−57.6 (−69.5, −38.9)
Interpersonal violence	457.5 (380.6–556.7)	−38.5 (−56.4, −15.8)	49.4 (37.9–63.7)	−17.1 (−20.2, −13.6)	408.1 (332.5–504.8)	−40.4 (−59.1, −15.7)
Conflict and terrorism	221.1 (124.5–440.2)	−96.4 (−98, −93.1)	212.3 (115.8–430.6)	−46.9 (−64.9, −12.7)	8.8 (8–9.7)	−99.8 (−99.9, −99.8)
Physical violence by firearm	132.8 (99.7–169.4)	−38.4 (−63.6, −6)	0.8 (0.6–1.1)	−23.4 (−25.7, −20.8)	132 (98.9–168.6)	−38.5 (−63.8, −5.8)
Executions and police conflict	8.8 (7.6–10.6)	1542.4 (1,115, 2046.8)	3.4 (2.3–5)	0 (0, 0)	5.4 (5–6)	912.5 (681.3, 1152.5)
Physical violence by other means	178.2 (138–217.9)	−41.5 (−61.9, −16.2)	13.7 (9.8–18.3)	−30 (−32.1, −28)	164.5 (124.5–205.3)	−42.3 (−63.9, −15.2)
Physical violence by sharp object	119.2 (91–151.8)	−38.9 (−60.6, −2.9)	7.5 (5.4–10)	−26.1 (−28.2, −24.3)	111.6 (84.5–145.4)	−39.6 (−62.1, −1)
Self-harms by firearm	19.9 (13.1–33.4)	−52.1 (−75.3, −9.1)	0.5 (0.3–0.6)	−21.3 (−24.2, −18.6)	19.5 (12.6–32.9)	−52.6 (−75.7, −8.8)
Self-harm by other specified means	313.1 (252.9–390.9)	−57.7 (−69.6, −39.8)	8 (5.7–10.5)	−46.6 (−48.9, −44)	305.2 (243.4–381.5)	−57.9 (−69.9, −39.7)

## Discussion

Our analysis incorporated the burden and trend of injuries associated with morbidity and mortality at the national and sub-national levels in Ethiopia from 1990 to 2019 using the GBD study data. While injury-related deaths have substantially decreased over the past three decades, they still account for 7.8% of deaths from all-causes. When comparing the burden of major public health priority diseases in Ethiopia, deaths due to injury are greater than the combined deaths from malaria and tuberculosis. The death rate has declined by 70% between 1990 and 2019. This may be related to indirect effects of Ethiopia’s overall socioeconomic improvement over the previous two decades, including access to health, education, and social services (29). There is good evidence that improved living conditions, reduced poverty, and better access to services are determinants of injury-related morbidity and mortality (30–33). The 2019 data are higher than a similar study conducted in 2017 (8), although within the margin of error. The results demonstrate that injuries are not declining quickly enough to be in line with the SDG target by 2030.

At sub-national level, this study has revealed that the Southern Nations Nationalities and Peoples’ region has the highest injury-related deaths and DALYs, followed by the Afar region. There is no comparable study in Ethiopia to compare the findings regionally. However, a systematic review and meta-analysis of road injuries has shown that the Southern Nations Nationalities and Peoples’ region has the highest disability associated with injury (21). There are several potential explanations for this. The region has a dense population, mobile youth, local and international trafficking, and high level of migration (34–36). Moreover, the region is comprised of more than 65% of all ethnic groups in Ethiopia, and this may be linked with a higher frequency of interpersonal violence related injuries. In terms of YLD and prevalence rate, the Gambella and Tigray regions were disproportionately affected. The Tigray region has experienced a prolonged conflict between armed groups and the government forces, the Ethio-Eritrean war, and frequent boarder conflicts with Eritrea (14, 16). However, the region has made relatively better progress in healthcare coverage, education, and infrastructure that could have decreased injury-related deaths and increased people living with long-term disability (37). The Gambella region also shares a similar history of intergroup conflicts and frequent short-term conflicts between the government forces and armed groups (14). The events in these regions may have contributed to the injury death rate being the highest in 1999 and 2000, specifically the war between Ethiopia and Eritrea (14, 16). However, all regions throughout the country have maintained a reduction of injury-related death and disability in the past 30 years. On the other hand, the absolute number of injuries did not decrease significantly and actually increased from 2010 to 2019. High developmental activities in the country in recent years and increased population size might have contributed to this trend (38), whereas an improvement in healthcare services may have improved the survival of individuals who experienced severe injuries.

Throughout the study period at the national and sub-national levels, males were disproportionately affected by injury. The literature shows that males are more likely to die and suffer long-term disability from injury than females (5, 39, 40). There are several possible reasons to explain men’s vulnerability to injuries in a patriarchal society, such as Ethiopia. Males tend to engage in risky behaviors, like drive over limit, non-use of seatbelts, drive after drinking alcohol, and unsafe jobs (7, 41, 42). They also engage in armed conflicts (42, 43). Moreover, studies indicate that males use illegal substances, drugs, and alcohol more often than females, this may increase the level of aggression, involvement in criminal activities, and injury risk (41, 42). When investigating the age distribution, the risk of death and disability from injuries was observed to be higher among the lower age groups. This could be explained by the fact that people in this age group are less likely to keep workplace safety precautions, have higher risk-taking behaviors, and engage in injury prone activities (41, 44, 45).

Approximately 79% of the total injury-related deaths were caused by the five most frequent injuries: road traffic injuries, interpersonal violence, self-harm, falls, and poisoning. The road traffic injuries as the leading causes of injury-related death and disability aligns closely with the global injury ranking of road injuries, self-harm, and interpersonal violence (6). Moreover, road traffic injuries account for more than 61% of injury-associated hospitalizations and 52% of these had fatal outcomes (7, 10, 11, 20). The Ethiopian health sector transformation plan recognizes that road traffic injuries are one of the growing and predominant public health problems in the country (37).

Interpersonal violence and self-harm are among the leading causes of premature deaths in Ethiopia. This is consistent with available surveillance data, which revealed that assault and intentional self-harm accounts each for 10% of overall injury-related deaths (12). Falls are becoming a main cause of injury resulting in death and disability in Ethiopia (12, 13). The present study showed that nationally falls are the fourth leading cause of death, and the numbers of deaths and DALYs associated with falls have increased by 55 and 46%, respectively; between 1990 and 2019. This is the only injury cause for which the rates of DALYs and deaths remained unchanged over time. This might be due to the increase of aging populations as a result of improved life expectancy in Ethiopia (38). Studies indicate that people older than 65 years may experience frequent falls that cause moderate to severe adverse health consequences (13).

The GBD methodologies’ limitations and strengths are discussed elsewhere in the literature, and need to be taken into account when interpreting the results (5, 6). Yet, this study has a number of strengths, including comprehensive and representativeness at sub-national level using all Ethiopian data sources that are readily available, which enhance the validity of estimates that cannot be seen in individual household surveys with varied data quality. However, the absence of a reliable injury surveillance and vital registration system in the country may lead to wider uncertainty in determining the causes of injury mortality.

### Injury prevention initiatives in Ethiopia

Preoccupied with communicable disease control, Ethiopia has long ignored injury, despite the fact that it imposes a significant economic and health burden on the country. However, recent years have seen a rise in focus on injury prevention (37, 46). Different government bodies, including the Ministry of Health, Ministry of Transport, and Ministry of Women and Child Affairs are working on at least one type of injury prevention, which may be strengthened as a result of the current study’s findings. For instance, the Ethiopian essential health services package includes emergency surgeries (46), which are crucial for addressing the effects of injuries, while the Ministry of Women and Children Affairs is working on preventing gender-based violence and providing victims with treatment. In addition, individual activists, opinion leaders, and well-known writers and musicians in Ethiopia continuously engage in safety advocacy, education, and injury prevention to dispel the widespread misconception of there is nothing that can be done to prevent injuries. Additionally, a local NGO by the name of Save The Nation focuses on capacity building, road safety awareness creation initiatives, media campaigns, and resource mobilization. In order to avert post-injury adverse outcomes, insurance agencies are deemed to cover the emergency healthcare costs. Therefore, this study enables the stakeholders working on injury prevention through evidence-based priority settings, resource mobilization, and advocacy efforts.

## Conclusion

In Ethiopia the number of deaths, age-standardized rates of incidence, prevalence, DALYs, YLLs, and YLDs due to injuries have decreased steadily during the past 30 years. Yet, the burden remains unacceptably high. Moreover, there is a substantial regional disparity in the burden of injury. The majority of deaths and disabilities are caused by traffic injuries, falls, self-harm, and interpersonal violence. Children under the age of 5 years, adolescents, and young adults are disproportionately affected by injuries. The majority of injuries are preventable using simple public health measures, road safety, and security interventions. Coordinated efforts are, therefore, required by researchers, public health authorities, and policymakers to reduce the burden of injury in Ethiopia.

## Data availability statement

The datasets presented in this article are not readily available because one can access them from the IHME repository directly. Requests to access the datasets should be directed to http://ghdx.647healthdata.org/gbd-results-tool.

## Ethics statement

Ethical review and approval was not required for the study on human participants in accordance with the local legislation and institutional requirements. Written informed consent from the participants’ legal guardian/next of kin was not required to participate in this study in accordance with the national legislation and the institutional requirements.

## Author contributions

TB conceived, designed, and conducted the study. ST, AM, and MS consulted the overall process of the study. ST, TB, AM, MS, FG, GT, SW, SMe, SMo, AW, and MN involved in the analysis and interpretation of the findings. All authors contributed to the article and approved the submitted version.

## Conflict of interest

The authors declare that the research was conducted in the absence of any commercial or financial relationships that could be construed as a potential conflict of interest.

## Publisher’s note

All claims expressed in this article are solely those of the authors and do not necessarily represent those of their affiliated organizations, or those of the publisher, the editors and the reviewers. Any product that may be evaluated in this article, or claim that may be made by its manufacturer, is not guaranteed or endorsed by the publisher.
